# Serum Aminoacyl-tRNA Synthetase-Interacting Multifunctional Protein-1 Can Predict Severe Antineutrophil Cytoplasmic Antibody-Associated Vasculitis: A Pilot Monocentric Study

**DOI:** 10.1155/2019/7508240

**Published:** 2019-05-20

**Authors:** Sung Soo Ahn, Jin-Ock Kim, Taejun Yoon, Jason Jungsik Song, Yong-Beom Park, Sang-Won Lee, Sang Gyu Park

**Affiliations:** ^1^Division of Rheumatology, Department of Internal Medicine, Yonsei University College of Medicine, Seoul, Republic of Korea; ^2^College of Pharmacy, Ajou University, Suwon, Gyeonggi-do, Republic of Korea; ^3^Institute for Immunology and Immunological Diseases, Yonsei University College of Medicine, Seoul, Republic of Korea

## Abstract

We investigated whether serum aminoacyl-tRNA synthetase-interacting multifunctional protein-1 (AIMP1) could predict severe cases of antineutrophil cytoplasmic antibody (ANCA)-associated vasculitis (AAV) based on the Birmingham vasculitis activity score (BVAS). Sixty-one patients with AAV were selected for inclusion from our prospective AAV cohort. AAV-specific indices and clinical manifestations were assessed, and laboratory tests were performed on the day of blood sampling. Patients with severe AAV were defined as those with a BVAS higher than the lower limit of the highest tertile of BVAS (BVAS ≥ 12). We measured serum AIMP1 levels of the stored serum samples. A total of 20 (32.8%) and 41 (67.2%) patients were classified as having severe and nonsevere AAV according to the cut-off of BVAS ≥ 12. Patients with severe AAV showed higher frequencies of general and renal manifestations, along with ANCA positivity, and exhibited a higher mean neutrophil count, erythrocyte sedimentation rate, and C-reactive protein levels, but lower mean haemoglobin and serum albumin levels than those with nonsevere AAV. The mean serum AIMP1 level in patients with severe AAV was significantly higher than that of patients with nonsevere AIMP1 (351.1 vs. 98.4 pg/mL, p = 0.006). Multivariate logistic regression analysis including variables showing significance in univariate analyses revealed that only serum AIMP1 exhibited a significant association with severe AAV (odds ratio 1.004, p = 0.031). When we set the optimal cut-off of serum AIMP1 for severe AAV to 50.28 pg/mL, patients with severe AAV more frequently had AIMP1 levels above the cut-off than those with nonsevere AAV (80.0% vs. 31.7%, relative risk 8.615, p < 0.001). The results from our study suggest that serum AIMP1 can be used to estimate the cross-sectional severe AAV population based on the BVAS.

## 1. Introduction

Antineutrophil cytoplasmic antibody (ANCA)-associated vasculitis (AAV) is characterized by necrotising vasculitis in small-sized vessels and is categorized into three variants: microscopic polyangiitis (MPA), granulomatosis with polyangiitis (GPA), and eosinophilic granulomatosis with polyangiitis (EGPA), based on the 2012 Chapel Hill Consensus Conferences (CHCC) Nomenclature of Vasculitis [1]. Activated macrophages secrete several proinflammatory cytokines such as tumour-necrosis factor (TNF)-*α*, interleukin (IL)-1*β*, and IL-6, which prime neutrophils to subsequently release myeloperoxidase (MPO) or proteinase 3 (PR3). The secreted MPO and PR3 can be recognized by antigen-presenting cells and presented to helper T cells, leading to the production of anti-MPO or anti-PR3 ANCAs by B cells [2]. ANCA-mediated activation of primed neutrophils provokes bulky inflammation on the vessel walls as well as in their adjacent tissues [3]. Thus, AAV may be triggered by both proinflammatory cytokines and autoreactive immune cells.

Aminoacyl-tRNA synthetase (ARS)-interacting multifunctional protein-1 (AIMP1) is one of the three nonenzymatic factors assembling a multi-tRNA synthetase complex with 11 different ARSs [4]. AIMP1 can also be secreted and may modulate the immune reaction by enhancing the production of proinflammatory cytokines such as TNF-*α*, IL-6, IL-8, and IL-12 by activated immune cells [5]. Thus, AIMP1 may participate in the pathogenesis of autoimmune diseases, especially AAV. We previously demonstrated the clinical potential of AIMP1 as a biomarker in patients with rheumatoid arthritis and systemic lupus erythematosus (SLE) [6, 7]. However, there is no report regarding the clinical role of serum AIMP1 in AAV. Hence, in this study, we investigated whether serum AIMP1 could be used to estimate the cross-sectional severity of AAV patients based on the Birmingham vasculitis activity score (BVAS) in a prospective cohort of AAV patients [8].

## 2. Materials and Methods

### 2.1. Patients, Clinical and Laboratory Data, and Serum AIMP1 Measurement

This study included 61 patients diagnosed with AAV selected among our prospective Severance Hospital ANCA-associated VasculitidEs (SHAVE) cohort. All patients were first diagnosed with AAV in the Department of Rheumatology, Yonsei University College of Medicine, Severance Hospital, from October 2000 to July 2018, and classified based on the 2007 European Medicines Agency algorithms and the 2012 CHCC Nomenclature of Vasculitis [1, 9]. On the day of blood sampling, patients with serious medical conditions other than AAV, such as serious infection or malignancies, were excluded from the study. This study was approved by the Institutional Review Board of Severance Hospital (4-2016-0901), and all patients provided written informed consent at the time of blood sampling.

On the day of blood sampling, the BVAS [8], five-factor score (FFS) (2009) [10], vasculitis damage index (VDI) [11], Korean version of the short form-36 (SF-36) [12], and clinical manifestations were assessed and laboratory tests were performed. Because the BVAS for GPA is determined according to a different weight system, we evenly applied the BVAS to patients with both MPA and GPA so as to unify the scoring system. We stratified AAV patients into three groups according to the tertile of BVAS, in which patients with a BVAS higher than the lower limit of the highest tertile (BVAS ≥ 12) were considered to have severe AAV. We also counted the number of patients under treatment with immunosuppressive drugs. We obtained whole blood samples from each patient with AAV, isolated the serum, and stored it at –80°C until analysis. AIMP1 levels were determined with human AIMP1 enzyme-linked immunoassay kits purchased from Cloud-Clone Corp. (Houston, TX, USA) according to the manufacturer's instructions [7].

### 2.2. Statistical Analyses

All statistical analyses were conducted using SPSS software (version 23 for windows; IBM Corp., Armonk, NY, USA). Differences in variables between the two groups (severe and nonsevere AAV) were analyzed using the chi-squared and Fisher's exact tests or the Mann-Whitney U test. The odds ratio (OR) was assessed using multivariate logistic regression analysis of variables with p-values less than 0.05 in univariate logistic regression analysis. The optimal cut-off of serum AIMP1 for severe AAV was extrapolated by constructing the receiver operator characteristic (ROC) curve. The relative risk of the optimal cut-off value of serum AIMP1 for severe AAV was analyzed using contingency tables and the chi-squared test. P-values less than 0.05 were considered statistically significant.

## 3. Results

### 3.1. Comparison of Clinical Characteristics in Patients with and without Severe AAV

The baseline characteristics of the 61 patients with AAV are described in Table 1, including 20 (32.8%) and 41 (67.2%) patients classified as having severe and nonsevere AAV, respectively. No significant differences were found concerning the variants of AAV and demographic data between the groups, except for the higher proportion of patients with new-onset AAV in the severe group (65.0% vs. 26.8%, p = 0.005). Patients with severe AAV exhibited higher mean BVAS and FFS (2009) than those without (p = 0.009). In addition, general and renal manifestations were more commonly observed and ANCA positivity was more often detected in patients with severe AAV. With respect to the routine laboratory results, patients with severe AAV exhibited a higher mean neutrophil count, erythrocyte sedimentation rate (ESR), and C-reactive protein (CRP) level, but showed lower mean haemoglobin and serum albumin levels than those without. There was also a lower proportion of patients on prednisolone treatment in the severe AAV group owing to the higher rate of new-onset AAV among these patients. The mean level of serum AIMP1 in patients with severe AAV was significantly higher than in those without (Table 1).

### 3.2. Logistic Regression Analysis

Baseline ANCA positivity and laboratory data with statistical significance were subsequently included in the univariate and multivariate logistic regression analysis. We excluded the FFS (2009) in the logistic regression analysis due to the similarity of its constituent items to those used to calculate the BVAS. In the multivariate logistic regression analysis, only serum AIMP1 exhibited a significant association with severe AAV (Table 2).

### 3.3. Optimal Cut-Off of Serum AIMP1 and Its Relative Risk for Severe AAV

**Figure 1:**
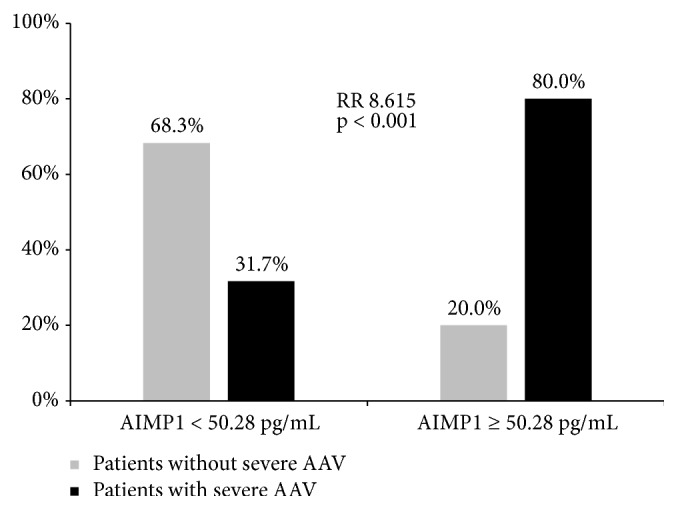
*Relative risk of severe AAV based on the optimal cut-off of serum AIMP1 (50.28 pg/mL)*. AAV: ANCA-associated vasculitis; AIMP1: aminoacyl-tRNA synthetase-interacting multifunctional protein-1; RR: relative risk.

The optimal cut-off level of serum AIMP1 for predicting severe AAV was determined to be 50.28 pg/mL based on the area under the ROC curve (0.757, 95% CI 0.625–0.890; sensitivity 0.800, specificity 0.683; Supplementary Figure S1). That is, severe AAV was more frequently identified in patients with a serum AIMP1 level ≥50.28 pg/mL than in those with a serum AIMP1 level <50.28 pg/mL (relative risk, 8.615; 95% CI: 2.400–30.923, p < 0.001; Figure 1).

## 4. Discussion

In this study, we investigated the clinical value of serum AIMP1 in stratifying AAV patients according to disease severity and demonstrated that serum AIMP1 could independently be used for cross-sectional classification of patients into severe and nonsevere AAV based on the BVAS.

Proinflammatory cytokines have been reported to be involved in the pathogenesis of AAV, including TNF-*α*, IL-1*β*, IL-6, IL-8, and IL-12 [7, 13]. Secreted AIMP1 was also reported to enhance the serum concentration of these cytokines [5]. Thus, this connection between the pathogenesis of AAV and the inflammatory action of secreted AIMP1 led us to speculate that serum AIMP1 would be a valuable marker to categorize patients at risk of severe AAV based on the BVAS. However, we could not clarify the direct correlation between serum AIMP1 and proinflammatory cytokines, as described in a previous report [13].

Despite substantial progress in understanding the pathogenesis of AAV, there is still no reliable biomarker to assess disease activity in AAV. As such, inflammatory factors such as ESR, CRP, and alterations of ANCA titres are still the most commonly used markers to assess disease activity in clinical practice. Here, we found that BVAS was significantly correlated with ESR, CRP, and AIMP1; however, the serum AIMP1 level was not correlated with ESR (p = 0.071) or CRP (p = 0.220). A previous study demonstrated that the correlation of major AAV-specific cytokines and ESR or CRP was relatively low [13]. Thus, we assume that serum AIMP1 could be used to estimate the cross-section of patients with severe AAV via intracellular signals, which could differ from the factors influencing ESR and CRP levels. Importantly, in this study, serum AIMP1 was significantly associated with severe AAV irrespective of ESR, CRP, and ANCA titres. Therefore, the clinical utility of serum AIMP1 could be emphasized by the fact that it could serve as a complementary index for predicting severe AAV, independently of ESR, CRP, and ANCA levels.

In our previous study, the cut-off level of serum AIMP1 for predicting active SLE was 10.09 ng/mL, which is about 200 times higher than the cut-off for predicting severe AAV determined in the present study (50.28 pg/mL). Furthermore, the mean serum AIMP1 level in patients with severe AAV was much lower than that in patients with active SLE (351.1 pg/mL vs. 8,000.0 pg/mL) [7]. Although we did not include patients with SLE in the present study, and thus could not directly compare serum AIMP1 between the two diseases, these results suggest that the dynamic range of the serum concentration of AIMP1 to estimate disease severity might be autoimmune disease-specific. Thus, the underlying autoimmune disease should be considered when determining cut-offs of serum AIMP1 to define high disease activity.

As a pilot study, this study has a strength in that it is the first demonstration of the clinical value of serum AIMP1 as a marker for cross-sectional analyses of severe and nonsevere AAV cases in the prospective SHAVE cohort, which includes clinical and laboratory data and blood samples collected on the same day. However, our study also has several limitations. First, the number of patients was not sufficiently large to represent the general Korean patient population with AAV or to conduct subgroup analyses due to the monocentric study design. Second, we could not serially measure serum AIMP1 levels in each AAV patient. Third, we could not clarify the correlation between serum AIMP1 and proinflammatory cytokines by measuring serum proinflammatory cytokine levels. Fourth, we were not able to measure serum AIMP1 levels in healthy subjects for comparison. Future studies with a larger number of AAV patients in the prospective SHAVE cohort and serially measured parameters will provide more reliable and validated information on the clinical implications of serum AIMP1 for stratifying AAV patients.

## 5. Conclusions

In conclusion, we have demonstrated that serum AIMP1 can be used as an estimate for cross-sectional analysis of severe AAV based on the BVAS. Thus, measuring serum AIMP1 might provide useful information regarding disease activity for monitoring patients with AAV.

## Figures and Tables

**Table 1:** Comparison of variables between patients with and without severe AAV.

	Patients with severe AAV (n=20)	Patients without severe AAV (n=41)	p-value
*Variants (N, (%))*			
MPA	12 (60.0)	20 (48.8)	0.414
GPA	7 (35.0)	11 (26.8)	0.515
EGPA	1 (5.0)	10 (24.4)	0.084
*Demographic data *			
Age (years)	63.2 ± 14.4	57.6 ± 14.0	0.153
Female gender (N, (%))	14 (70.0)	26 (63.4)	0.614
Disease duration (months)	10.8 ± 28.3	27.5 ± 47.8	0.093
New onset AAV	13 (65.0)	11 (26.8)	0.005
*AAV-specific indices*			
BVAS	17.4 ± 4.1	4.9 ± 3.0	<0.001
FFS (2009)	1.8 ± 1.0	1.1 ± 0.9	0.009
VDI	3.4 ± 1.5	3.0 ± 1.8	0.419
SF-36 PCS score	43.1 ± 20.0	53.3 ± 23.3	0.097
SF-36 MCS score	55.1 ± 19.4	59.3 ± 20.7	0.457
*Clinical features (N, (*%*))*			
General	10 (50.0)	7 (17.1)	0.008
Cutaneous	2 (10.0)	5 (12.2)	0.999
Mucous membrane and eye	1 (5.0)	2 (4.9)	0.999
Ear, nose, and throat	8 (40.0)	17 (41.5)	0.914
Pulmonary	16 (80.0)	22 (53.7)	0.055
Cardiovascular	1 (5.0)	2 (4.9)	0.999
Abdominal	1 (5.0)	0 (0.0)	0.328
Renal	17 (85.0)	17 (41.5)	0.002
Nervous system	4 (20.0)	8 (19.5)	0.999
*ANCA positivity (N, (*%*))*			
ANCA double positive	0 (0.0)	1 (2.4)	0.999
MPO-ANCA or P-ANCA positive	16 (80.0)	21 (51.2)	0.050
PR3-ANCA or C-ANCA positive	2 (10.0)	5 (12.2)	0.999
ANCA positivity	18 (90.0)	25 (61.0)	0.020
MPO titre (Units/mL)	52.8 ± 49.7	23.1 ± 41.6	0.017
PR3 titre (Units/mL)	1.6 ± 4.8	1.0 ± 4.3	0.637
*Laboratory data *			
White blood cell count (/mm^3^)	9,437.5 ± 4,008.4	7,733.9 ± 3,367.8	0.087
Neutrophil count (/mm^3^)	7,763.5 ± 3,642.9	5,196.5 ± 3,065.2	0.006
Haemoglobin (g/dL)	10.0 ± 2.2	12.6 ± 1.7	<0.001
Platelet count (× 1,000/mm^3^)	299.7 ± 129.5	271.5 ± 79.1	0.379
Creatinine (mg/dL)	2.4 ± 1.8	1.5 ± 1.9	0.093
Serum albumin (g/dL)	3.3 ± 0.6	4.0 ± 0.5	<0.001
AST (IU/L)	19.4 ± 7.9	22.5 ± 16.9	0.331
ALT (IU/L)	23.0 ± 14.6	20.8 ± 13.6	0.575
ESR (mm/hr)	55.5 ± 44.5	32.9 ± 21.5	0.042
CRP (mg/L)	29.0 ± 41.4	3.5 ± 5.7	0.013
*Immunosuppressive drugs (N, (*%*))*			
Prednisolone	10 (50.0)	33 (80.5)	0.014
Cyclophosphamide	4 (20.0)	5 (12.2)	0.420
Rituximab	1 (5.0)	0 (0)	0.149
Azathioprine	2 (10.0)	12 (29.3)	0.093
Tacrolimus	0 (0)	3 (7.3)	0.215
Mycophenolate mofetil	0 (0)	2 (4.9)	0.315
Methotrexate	1 (5.0)	1 (2.4)	0.598
*Novel serum biomarker*			
Serum AIMP1 (ng/mL)	351.1 ± 350.9	98.4 ± 67.9	0.006

Values are expressed as mean ± standard deviation or number (percentages).

AAV: ANCA-associated vasculitis; ANCA: antineutrophil cytoplasmic antibody; MPA: microscopic polyangiitis; GPA: granulomatosis with polyangiitis; EGPA: eosinophilic granulomatosis with polyangiitis; BVAS: Birmingham vasculitis activity score; FFS: five-factor score; VDI: vasculitis damage index; SF-36: short form-36; PCS: physical component summary; MCS: mental component summary; MPO: myeloperoxidase; P: perinuclear; PR3: proteinase 3; C: cytoplasmic; AST: aspartate aminotransferase; ALT: alanine aminotransferase; ESR: erythrocyte sedimentation rate; CRP: C-reactive protein; AIMP1: aminoacyl-tRNA synthetase-interacting multifunctional protein-1.

**Table 2:** Univariate and multivariate logistic regression analyses of laboratory variables with significance for prediction of severe AAV.

	Univariate analysis			Multivariate analysis		
	Odds ratio	95% CI	p-value	Odds ratio	95% CI	p-value
Laboratory variables						
ANCA positivity	5.760	1.175, 28.244	0.031	1.075	0.137, 8.416	0.945
Neutrophil count	1.000	1.000, 1.000	0.010	1.000	1.000, 1.000	0.967
Haemoglobin	0.511	0.360, 0.726	<0.001	0.616	0.368, 1.029	0.064
Serum albumin	0.069	0.015, 0.315	0.001	0.349	0.038, 3.203	0.352
ESR	1.022	1.004, 1.040	0.016	0.981	0.947, 1.015	0.272
CRP	1.070	1.005, 1.139	0.034	1.087	0.955, 1.238	0.207
Serum AIMP1	1.004	1.001, 1.006	0.003	1.004	1.000, 1.008	0.031

AAV: ANCA-associated vasculitis; ANCA: antineutrophil cytoplasmic antibody; CI: confidence interval; ESR: erythrocyte sedimentation rate; CRP: C-reactive protein; AIMP1: aminoacyl-tRNA synthetase-interacting multifunctional protein-1.

## Data Availability

The data used to support the findings of this study are available from the corresponding author upon request.

## Supplementary Materials

Supplementary Materials Figure S1. Receiver operator characteristic curve of serum AIMP1 to discriminate between presence and absence of severe AAV. AIMP1: aminoacyl-tRNA synthetase-interacting multifunctional protein-1; AAV: ANCA-associated vasculitis; ANCA: antineutrophil cytoplasmic antibody; AUC: area under the curve; CI: confidence interval.
