# Supplementary figures and images for: Serum Aminoacyl-tRNA Synthetase-Interacting Multifunctional Protein-1 Can Predict Severe Antineutrophil Cytoplasmic Antibody-Associated Vasculitis: A Pilot Monocentric Study
Source: Biomed Res Int. 2019 May 20;2019:7508240. doi: 10.1155/2019/7508240 (PMC6545776; doi:10.1155/2019/7508240)

## Slide 1
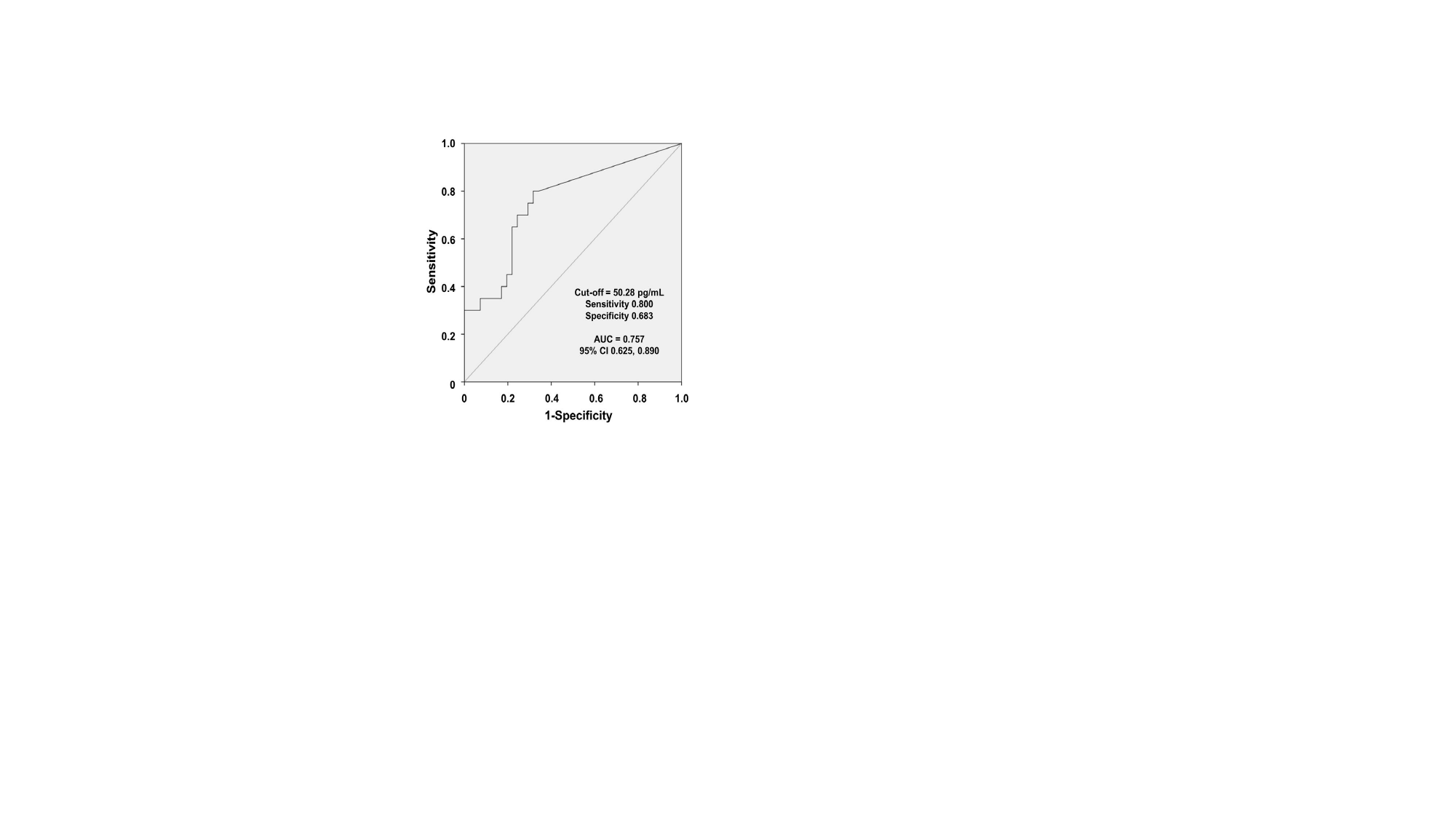

Supplement: Supplementary Materials — Figure S1. Receiver operator characteristic curve of serum AIMP1 to discriminate between presence and absence of severe AAV. AIMP1: aminoacyl-tRNA synthetase-interacting multifunctional protein-1; AAV: ANCA-associated vasculitis; ANCA: antineutrophil cytoplasmic antibody; AUC: area under the curve; CI: confidence interval. [file 7508240.f1.pptx]
